# Histological Aspects and Quantitative Assessment of Ki67 as Prognostic Factors in Breast Cancer Patients: Result from a Single-Center, Cross Sectional Study

**DOI:** 10.3390/medicina56110600

**Published:** 2020-11-09

**Authors:** Irina Niță, Cornelia Nițipir, Ștefania Andreea Toma, Alexandra Maria Limbău, Edvina Pîrvu, Ioana Anca Bădărău, Ioana Suciu, George Suciu, Loredana Sabina Cornelia Manolescu

**Affiliations:** 1 Faculty of Medicine, Faculty of Midwifery and Nursing, Carol Davila University of Medicine and Pharmacy, 020021 Bucharest, Romania; nitipir2003@yahoo.com (C.N.); ancab52@yahoo.com (I.A.B.); 2 Clinic of Oncology, Elias Universitary Emergency Hospital, 011461 Bucharest, Romania; 3 Medical Oncology Department, Ponderas Academic Hospital, 014142 Bucharest, Romania; stefaniaandreeachivu@yahoo.com; 4 Dermatology Department, Municipal Hospital Curtea de Argeș, 115300 Curtea de Argeș, Romania; alexandra_021286@yahoo.fr; 5 Medical Oncology Department, Clinical Hospital Colţea, 927180 Bucharest, Romania; edvinapirvu@gmail.com; 6 BEIA consult International, Peroni 16, 041386 Bucharest, Romania; ioana.suciu@beia.ro (I.S.); george@beia.ro (G.S.)

**Keywords:** Ki67, histologic grade, breast cancer, histologic type, progression-free survival, overall survival

## Abstract

*Background and objectives:* Our aim is to explore the relationship between the levels of protein encoded by Ki67 and the histopathological aspects regarding the overall survival and progression-free survival in a single university center. A secondary objective was to examine other factors that can influence these endpoints. New approaches to the prognostic assessment of breast cancer have come from molecular profiling studies. Ki67 is a nuclear protein associated with cell proliferation. Together with the histological type and tumor grade, it is used to appreciate the aggressiveness of the breast tumors. *Materials and Methods:* We conducted a retrospective single-institution study, at Elias University Emergency Hospital, Bucharest, Romania, in which we enrolled women with stage I to III breast cancer. The protocol was amended to include the immunohistochemistry determination of Ki67 and the histological aspects. The methodology consisted in using a Kaplan–Meier analysis for the entire sample and restricted mean survival time up to 36 months. *Results:* Both lower Ki67 and low tumor grade are associated with better prognosis in terms of overall survival (OS) and progression-free survival (PFS) for our patients’ cohort. In our group, the histological type did not impact the time to progression or survival. *Conclusions:* Both overall survival and progression-free survival may be influenced by the higher value of Ki67 and less differentiated tumors. Further studies are needed in order to establish if the histologic type may impact breast cancer prognostic, probably together with other histologic and molecular markers.

## 1. Introduction

Breast cancer (BC) is the most frequent malignancy in women worldwide and the leading cause of cancer death in young women. Over the past 10–15 years, treatment concepts changed radically due to new molecular heterogeneity of the tumors [1]. Nowadays, clinical research emphasizes more on biologically directed therapy and immunotherapy. On the other hand, the routinely molecular testing had begun a driving principle of modern-day treatments with high impact in both locoregional tumor burden and systemic treatment [2,3].

The two major pillars of breast cancer management are locoregional treatment and systemic therapy, both guided by the histological and molecular tumor characteristics. The intrinsic classification of Perou and Sorlie, reported in 2000, distinguished four subtypes of breast cancer: luminal A and luminal B (expressing the estrogen receptor (ER)), basal-like and human epidermal growth factor receptor 2 (HER2) enriched subtype (without ER expression). This classification shifted clinical management of breast cancer from being based on tumor burden to biology-centered approaches. Currently, a clinical practice typically uses a surrogate classification of five subtypes based on histological and molecular characteristics, taking into account the expression of Ki67 [4,5,6,7,8]. Prognostic factors are used for the estimation of how aggressive the tumor may evolve and for the decision of treatment approach. A very important aspect is the performance status for each patient, together with other comorbidities. It is very important to take into consideration all the clinical history of each patient in order to prevent the complications of chemotherapy, but also to offer the best treatment to patients with pre-existing disease [9,10,11,12,13,14].

Metastasis is the principal cause of breast cancer death, with this event appeared in probably 20–30% of patients with early breast cancer. The risk of recurrence is, at the same time, affected by the stage at the initial presentation and the biological features of the tumor. The histological aspects together with the Ki67 assessment are used for estimation of prognosis and guiding the decision on treatment choice [15,16,17].

The identification of appropriate patients for adjuvant BC therapies is still a challenge for medical oncologists. Tumor proliferation is a cornerstone of cancer progression and the use of Ki67 is an important aspect, but cell- cycle associated biomarkers, such cyclin D1, cyclin E and p21, considered, as well as prognostic factors, may be associated with a good treatment choice [18,19].

### 1.1. Ki67

#### 1.1.1. Structure and Function of Ki67

Ki67 is an antigen described for the first time in the 1980s by Gerdes et al. in mice and finally, in 1991, identified in humans. The gene of Ki67 is located on the long arm of human chromosome 10 and is composed of 15 exones and 14 introns. The cellular location is cell cycle-dependent, which means it is no expressed during the resting phase G0, levels are low during the G1 and early phase and rise in mitosis, thus a significant correlation between the proliferation index and mitotic activity [20,21,22].

#### 1.1.2. Detection of Ki67

The antigen Ki67 is detected in paraffin-embedded samples by the antibodies MIB-1 and MIB-3. The first one seems to be superior for assessing cycling cells on fixed and processed material because it has a good correlation with Ki67 expression on frozen material [23].

Initially, the scoring system is explained in the percentage of tumor cells stained by the antibody. Nowadays, automated readers are used in scoring large samples. The advantage of this method is that it might be used on fine needle biopsies, but can also count nonmalignant nuclei and cause a false positive result. Especially for breast cancer, tissue microarray technology has been used only for primary tumor samples, nor from axillary lymph node metastases or from malign tissue after neoadjuvant chemotherapy [24,25].

#### 1.1.3. Ki67 Cut-Off

The Ki67 cut-off was a continuous debate over time, due to the analytic barriers and the continuous distribution of the variable, consistent data, deriving from the context of trials that included BC patients.

A single mono-institutional study suggested that Ki67 can significantly discriminate the prognosis of patients with invasive lobular carcinoma, which suggests that this prognostic factor should be assessed regarding other tumor characteristics [26,27].

For a practical purpose, in order to treatment decision-making, the clinicians must distinguish between “luminal A” (more endocrine- sensitive, more indolent, better prognosis) and “luminal B” Her negative” (less endocrine-sensitive, more aggressive and worse prognostic) BC subtypes. Ki67 may be used in addition to estrogen and progesterone receptors for the application of this classification [28].

At the St. Gallen Consensus Conference, most of the expert panel voted that Ki-67 status should be defined as “high” or “low”, the threshold of ≥ 20% should be defined as “high” Ki-67 status. The European Society of Medical Oncology recommended using the 20% cut-off value, with the mention that quality assurance programs are essential for laboratories reporting these results [29,30].

### 1.2. Histological Type and Grade

Breast cancer can be classified into biologically and clinically meaningful subgroups according to tumor grade and histological type. The grade is an assessment of the degree of differentiation (i.e., tubule formation, nuclear pleomorphism and proliferative activity (i.e., mitotic index)) of a tumor and reveals its aggressiveness. Tumor grade has been incorporated in multiple algorithms to determine breast cancer therapy. In breast cancer, the tumor grade is divided in three categories: G1—low/well-differenced grade; G2—medium grade; and G3—high histologic grade [16,31,32].

Histological type refers to the growth pattern of the tumors. The most frequent type of breast carcinoma is the ductal carcinoma not otherwise specified or of no special type. The special type includes up to 25% of breast cancer and encounter more than 17 distinct histological types. The most frequent of those special types is lobular carcinoma [33].

Our aim is to explore the relationship between the levels of protein encoded by Ki67 and the histopathological aspects regarding the overall survival and progression-free survival in a single university center. A secondary objective was to examine other factors that can influence these endpoints.

## 2. Materials and Methods

We conducted a retrospective, observational study on the whole group of patients with breast cancer treated in Elias Emergency Hospital Bucharest, Romania, between January 2014 and December 2019. The research was undertaken with the approval of the Elias University Hospital Ethics Committee (identification code 5925, date of approval 24 August 2018).

The including criteria for this trial were: age (>18 years), early and advanced invasive breast cancer, ECOG (Eastern Cooperative Oncology Group) performance status of a maximum of one, unilateral breast tumor and absence of pregnancy in the last 6 months. The principal exclusion criteria were the presence of metastases detected at the first CT scan.

Data regarding clinical examination with local evaluation, imagistic examination, histopathologic and immunohistochemistry exam were collected at baseline and reviewed at each subsequent visit. Follow-up visits were scheduled every 3 months and all patients were followed for a maximum of 36 months.

The samples were obtained from either needle core biopsy or radical mastectomy or lumpectomy, both before chemotherapy and were fixed in 10% buffered formalin, paraffin-embedded and stained with Hematoxylin–Eosin for histopathological examination. The cold ischemic time prior to excision was no longer than one hour. The needle cores were formalin-fixed immediately after extraction; the fixation times ranged from 24 to 72 h. All available hematoxylin and eosin (HE)-stained slides were reviewed. For immunohistochemical studies, paraffin-embedded tissue blocks from all patients were cut at 3-μm thick sections, placed on slides, deparaffinized in xylene and hydrated in a decreasing ethanol series.

### 2.1. Ki67 Assessed

The Ki67 (MIB-1 clone) percentage was assigned with immunohistochemistry, respecting all producers’ protocols. First, Hematoxylin–Eosin stains were examined at ×2 and ×10 magnification to identify cancerous regions within a tissue sample. Second, the MIB-1 clone stain for Ki67 was examined at ×2 and ×10 magnification to identify hot spots, i.e., areas with an increased number of Ki67-positive cells within the previously identified cancerous regions. Finally, using ×40 magnification over the hot spot, 10 cancer cells at a time were evaluated. Nuclei browner than blue were scored positive. The number of Ki67-positive tumor cells from each set of 10 was recorded. The field of magnification was divided visually into eight “pie slices” that were evaluated from the center of the field towards the outer edge. When the entire field of magnification did not include enough cancer cells, a new field was chosen, often within the same hot spot and adjacent to the original field. If no initial hot spot could be discerned, a new field was chosen at random. Each core biopsy and surgical sample was evaluated by a single observer (QR) with the observer blinded to the relationships between the samples.

Because the range of Ki67 was between <10% to 90%, we decided to divide into categories based on the cut-off value: “Ki67 low”—values less than 52.50, with a proportion of 80% of all patients; and “Ki67 high”—values greater than or equal to 52.50, being represented by 20% of patients.

### 2.2. Statistical Analysis

For statistical analysis, we used the R program, version 4.0.2. and the survminer package. The sensitivity level was 95%, with *p* < 0.05, considered statistically significant.

For the statistical analysis, we had two principal endpoints: overall survival (OS) and progression-free survival (PFS) considering Ki67, histological type and grade. OS was measured from the date of surgery or biopsy to the date of death or up to 36 months. PFS was measured from the date of surgery or biopsy until the first local or distant recurrence.

Our research was carried out with the approval and in accordance with the guidelines of the local Ethics Committee. All the procedures in the study respect the ethical standards in the Helsinki Declaration, and the protocol was approved by the Ethics Committee with the code 7423/2018 and 223/2019.

## 3. Results

One hundred and forty-three patients newly diagnosed with breast cancer without metastasis were recruited in our hospital. The mean age was 52 (range 27–78, SD = 12). We divided the statistical analysis into two chapters according to the established endpoints, OS and PFS. These endpoints were calculated for Ki67, tumor grade and histological type for all the patients admitted to our clinic over a 6-year period.

Both OS and PFS were measured using restricted mean survival time (RMST). RMST evaluated a time-to-event outcome, illustrated survival functions, and conventionally reported an alternative to hazard ratios to express the magnitude of the treatment effect when comparing between groups. Due to this short follow-up period, 36 months, the median survival could not be calculated [34].

### 3.1. OS Analysis

#### 3.1.1. Ki67 Analysis

In Table 1 we analyzed the expression of Ki67 (%) in all patients, irrespective of the molecular characteristics. In this cohort, we observe that an increase with 1% of the Ki67 expression correlated with an increase of death hazard ratio with 2%, statistically significant (*p* = 0.02), hazard ratio (HR) = 1.02 and confidence interval (CI) = 1.01 − 1.04. 

**Figure 1:**
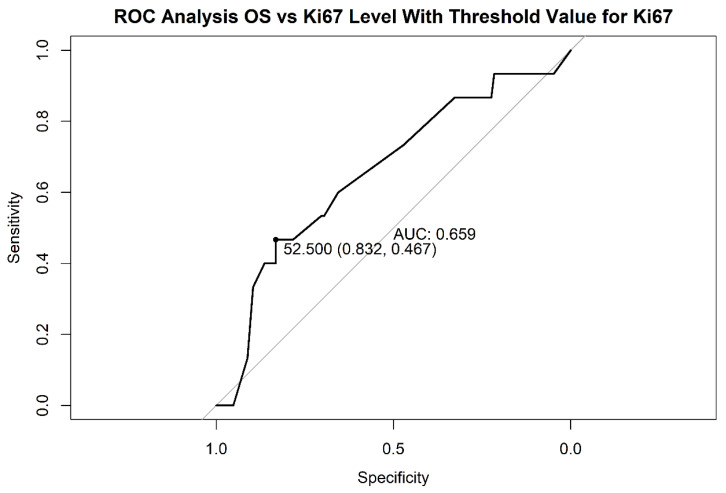
The ROC analysis vs. ki67 level. ROC: receiver operating characteristic, AUC: area under curve, OS: overall survival.

We determined the cut-off grade with the receiver operating characteristic (ROC) analysis and we observed that the area under the curve is 0.66 and the cut-off value, calculated with the Youden method, was 52.50, with a 0.83 sensibility and 0.46 specificity (Figure 1). The patients with low Ki67 had a better OS, the effect is statically significant (the log-rank test: χ^2^ = 8.30, degrees of freedom = 1, *p* = 0.0040)

**Figure 2:**
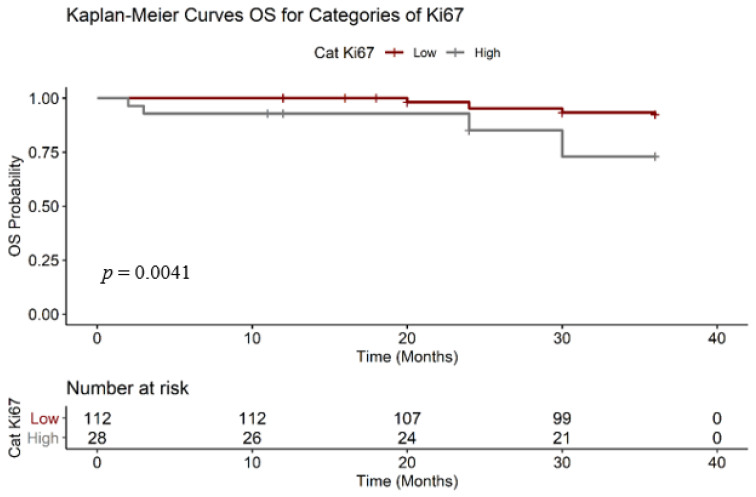
Overall survival (OS) Ki67 Kaplan–Meier.

To estimate the survival function, we analyzed the two Ki67 categories with RMST (Figure 2), Ki67 low (*n* = 112) and Ki67 high (*n* = 28). We observed that a 1% increase in the expression of Ki67 was correlated with an increase in the risk of death ratio, with the effect statistically significant (*p* < 0.05). As shown in Table 2 the RMST OS for the group with “low” KI67 is 35.20 months, compared with 31.90 months of patients with “high” Ki67.

#### 3.1.2. Histological Type

**Figure 3:**
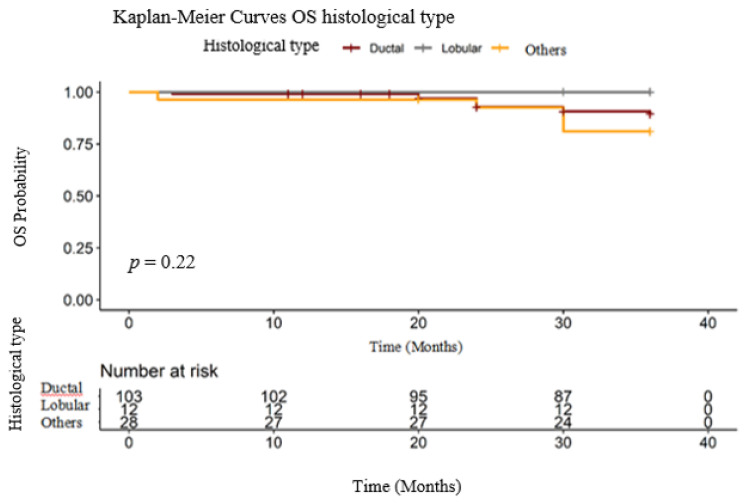
OS histological type.

In our cohort, 103 patients had ductal carcinoma/ no specific type (NST), 12 lobular carcinoma and 28 other types of breast cancer histology (20 patients with mixed type- NST and invasive lobular carcinoma, 4 with metaplastic carcinoma, 2 with medullary carcinoma and 2 with invasive papillary carcinoma). As we can see in Figure 3, in our group, there were no statistical differences in OS for these patients. Even more, we can see that in the lobular subgroup no patient developed metastatic disease. Of 20% of patients presented with other histological types, included mucinous (colloid) *n* = 15, medullary BC-7 and metaplastic BC *n* = 6 (Table 3).

#### 3.1.3. Tumor Grade

**Figure 4:**
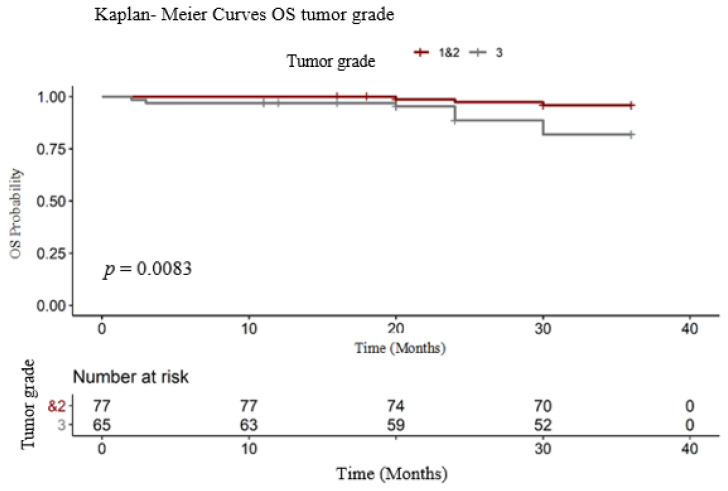
OS tumor grade.

In order to determine if the tumor grade can play a role in prolonging OS, the group was divided in two subgroups. The first one consisted of 77 patients (histologic grade 1 (*n* = 8) and 2 (*n* = 69) and the other consisted of grade 3 (*n* = 65). In our group of patients (Figure 4), both the RMST and the event percentage were significantly lower in patients with high-grade tumors (long-rank test: χ^2^ = 7.00, degrees of freedom = 1, *p* = 0.0083). The event (death) was present in 3.89% of patients with G1 and G2 and in 16.92 % of patients with G3 BC tumors (Table 4). The RMST OS for patients with low histologic grade (G1 and G2) was 35.50 months, compared with patients with high tumor grade (G3), 33.50 months.

### 3.2. PFS Analysis

#### 3.2.1. Ki67 Analysis

**Figure 5:**
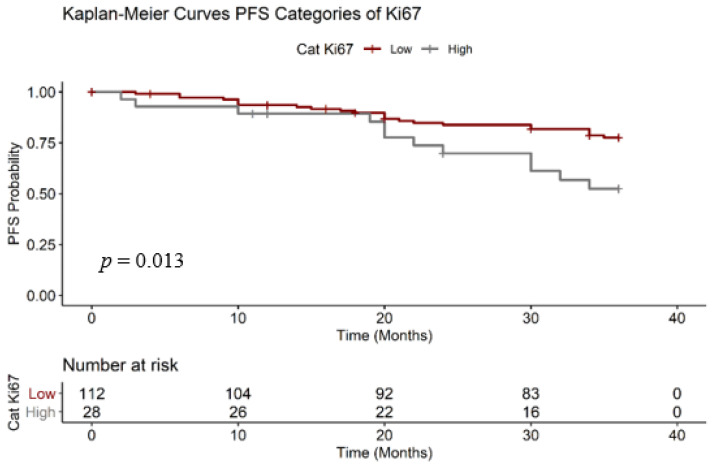
Progression-free survival (PFS) Ki67 Kaplan–Meier.

We also estimated the PFS for the two Ki67 subcategories (Figure 5). Like OS, PFS was also statistically significant, with *p* = 0.0130. We calculated the PFS considering the presence of imagistic metastasis disease. In Table 5 we calculated the RMST PFS of patients with “low” ki67 was 32.30 months, longer than the group “high” Ki67 (29.00 months).

#### 3.2.2. Histological Type

Regarding the PFS, histological type did not impact the OS (long- rank test: χ^2^ = 4.90, degrees of freedom = 1, *p* = 0.0083).

#### 3.2.3. Tumor Grade

**Figure 6:**
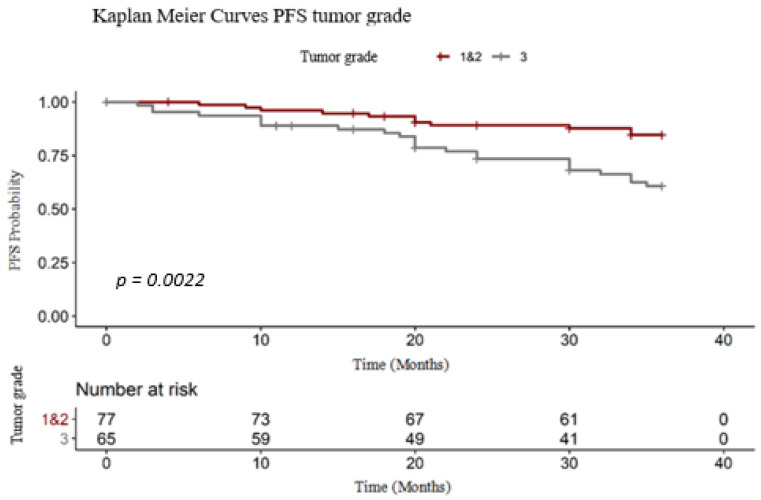
PFS tumor grade Kaplan–Meier.

Like OS, tumor grade which also influenced the PFS (Figure 6). Patients with G3 tumor grade had progressed faster than the other, with a shorter PFS (long-rank test: χ^2^ = 9.30, degrees of freedom = 1, *p* = 0.0022). The PFS RMST was 35.50 for G1 and G2 grade tumors and 29.80 months for high-grade tumors (Table 6).

## 4. Discussion

In our institution, before receiving any oncologic treatment, all the patients benefited from both an accurate staging of the disease and a histological and molecular description of the tumor. During the chemotherapy treatment, we performed a clinical examination for every patient at each visit, as well as an imaging evaluation every three months. With this approach, we wanted to encounter treatment-related complications, but also to detect the presence of the metastatic disease early. Medical Internet of Things (IoT) can be used for continuous telemetry, feeding real-time data into predictive modeling for early warning systems, especially in case of pandemic crises when access to medical facilities is difficult [35].

A potential limitation of this study is the absence of the same treatment for this cohort, including the fact that treatment algorithms are likely to have been inconsistent over the six years’ time frame of this study. Each patient that took part in the study was approved to be treated by a multidisciplinary team and received the treatment according to the stage of the disease and the tumor subtype. All therapies were given under international guidance consultation. In addition, we had some patients with delayed scheduled treatment, so with less dose intensity, and also some patients with adverse events (anemia, febrile neutropenia, hypersensitivity reactions) for which it was necessary to postpone the treatment, to adjunct chemotherapy treatment or to interrupt it.

PFS and OS between groups of patients with different levels of each biomarker have been compared using a log-rank test with a 5% level of statistical significance. A quantitative comparison based on the RMST was performed, which intuitively reflects the spatially varying effects of those prognostic factors.

Although the clinical applicability of this marker is not debatable, its use in pre-clinical practice may hinder its possible variation of results. Tissue type, warm and cold ischemic time, fixation medium and fixation time are examples of preanalytical variables. Antibody choice, scoring method or reporting strategy are examples of analytical and postanalytical variables. In this study, these variables were the same for the entire cohort and we focused on the survival impact of this prognostic factor [25,36,37].

Ki67 is an important molecular factor that can predict both overall survival (OS) and progression-free survival (PFS). This predictive marker, correlated with others like clinical study, hormonal receptors or histological tumor grade, is generally associated with prognostic significance in breast cancer patients [6].

Guidelines include a high Ki67 level as an indicator for increased risk of recurrence in patients who have estrogen receptor-positive, HER-2-receptor negative breast cancer patients. This aspect may conduct the clinicians to decide if the patient is a candidate for endocrine or chemotherapy [33]. In our study, according to the Log-rank test, the cell proliferation index (Ki67) has been proven to be statistically significant for the OS (*p*-value = 0.0268) and for the PFS (*p*-value = 0.0130), which means that the survival is statistically significantly influenced by this immunohistochemistry marker. The RMST difference between the two groups was 3.3 months for both OS and PFS.

In our study, we observed that an increase of 1% of the Ki67 expression correlated with an increase of the death hazard ratio with 2%, statistically significant (*p* = 0.02). This aspect must be taken into consideration especially for the classification of luminal tumors. Attention to the cut-off value was a point of interest for other authors, as well. In their study, published this year, Maltoni et Al., showed that the cut-off value of Ki67 is very important and must be updated, together with the detection method, in order to use the same antibody clones, platforms and scoring methods. The authors considered it irrelevant, for that we can consider the 10% cut-off value as a prognostic factor only under low PgR expression level in early BC [38].

We also analyzed some histopathologic aspects, such as histological type and tumor grade. Regarding the histological type, in our study, 72% of the patients presented ductal/NOS carcinoma, 8.3% presented lobular invasive subtype and 19.7% presented other types of histology. Regarding the tumor subtype, a potential limitation of this study is the lobular subtype known to be often associated with multicentricity and bilaterally, but also associated with longer survival, after considering tumor stage and size. In our study, we included only patients with unilateral breast cancer and one unique tumor after specific imagistic diagnosis [39].

Regarding the tumor grade, the group was divided into two subgroups, in the first one we included the G1 and G2 tumors with a total of 77 patients and in the other, G3 tumors, we included 65 patients. In our study, the lowest tumor grade is statistically correlated with prolonged OS and PFS, with the survival outcome, calculated with RMST, 2 months OS prolonged for the G1 and G2 group and 3.7 months for PFS.

We noticed a greater distribution of the patients with high-grade tumors (*n* = 65) and less common histologic types, in about 20% of the patients. The increased addressability of patients with aggressive tumors can be justified by the fact that those patients require aggressive treatment, careful monitoring of possible side effects due to neoadjuvant or adjuvant treatment, but also careful follow- up. In our country, those patients are redirected to oncology hospitals with multiple medical specialties, the final target being to maintain the dose intensity, to avoid delay and to detect metastatic diseases early [40].

Those three tumor markers were detected and quantified for all patients who came to our clinic for a period of 6 years, of course after meeting the inclusion criteria. The data presented in this study attest that there is a concordance between the practice of our institution and the data from the literature, as well as in concordance with the international guidelines. This study can be a reference model for all the Romania oncologic centers, given the fact that our hospital is in the first category and with a high addressability.

While our study has the potential limitation of being retrospective and having a relatively small number of subjects, it has the advantage of consistency in immunostaining results. Using both biopsy and mastectomy specimens, that had been immediately fixed after sample collection, all processing steps and evaluations were performed in a single institution, an important feature of our study given that inconsistencies in the assessment of Ki67 among institutions have yet to be resolved. Another important aspect is that the specimens were collected before receiving any oncological treatments.

## 5. Conclusions

In conclusion, both overall survival and progression-free survival may be influenced by the higher value of Ki67 and less differentiated tumors. The methodology was based on Kaplan–Meier analysis for the entire sample and RMST to evaluate a time--to--event outcome. Further studies are needed in order to establish if the histologic type may impact breast cancer prognostic, probably together with other histologic and molecular markers.

Our study verified that the low expression of Ki67 is associated with prolonged survival and an increase with 1% in the expression of Ki67 may lead to disease recurrence. OS and PFS RMST were both influenced by high levels of KI67 and high-grade tumors.

In the immunotherapy era, the proliferation rate is still an important factor to define the decision- making of adjuvant therapies. Ki67 is still a good marker, but aspects related to detection and scoring need to be improved, as well as correlations with the new molecular characteristics of the breast malignant cell (PIK3CA mutation, PD-L1 expression or BRCA mutation).

## Figures and Tables

**Table 1:** Distribution of Ki67.

Predictor	Coefficient	*p* Value	HR [CI95%]
**Ki 67**	0.02	0.0268	1.02 [1.01–1.04]

HR: hazard ratio, CI: confidence interval.

**Table 2:** OS Ki67 survival data.

Strata	Events (%)	RMST OS	Median Survival OS [CI95%]
**Ki67 Low**	7.14	35.20	N/A
**Ki67 High**	25.00	31.90	N/A

N/A: not applicable, RMST: restricted mean survival time.

**Table 3:** OS histological type.

Strata	Events (%)	RMST OS	Median Survival [CI95%]
**NST subtype**	9.70	34.70	N/A
**Lobular carcinoma**	0.00	36.00	N/A
**Others**	17.85	33.60	N/A

NST: no specific type.

**Table 4:** OS tumor grade.

Strata	Events (%)	RMST OS	Median Survival OS [CI95%]
**G1 and G2**	3.89	35.50	N/A
**G3**	16.92	33.50	N/A

**Table 5:** PFS Ki67 survival data. PFS: Progression-free survival

Strata	Events (%)	RMST PFS	Median Survival PFS [CI95%]
**Ki67 Low**	20.53	32.30	N/A
**Ki67 High**	42.85	29.00	N/A [30.00 la N/A]

**Table 6:** Tumor grade survival data.

Strata	Events (%)	RMST PFS	Median Survival PFS [CI95%]
**G1 and G2**	14.28	33.50	N/A
**G3**	35.38	29.80	N/A
